# Individual differences in the efficacy of a short theory of mind intervention for children with autism spectrum disorder: a randomized controlled trial

**DOI:** 10.1186/1745-6215-13-206

**Published:** 2012-11-09

**Authors:** Elske Hoddenbach, Hans M Koot, Pamela Clifford, Carolien Gevers, Cassandra Clauser, Frits Boer, Sander Begeer

**Affiliations:** 1 VU University Amsterdam, Amsterdam, Netherlands; 2 Wei43, Amsterdam, Netherlands; 3 De Bascule, Amsterdam, Netherlands; 4 AMC De Bacule, Amsterdam, Netherlands; 5 School of Psychology, University of Sydney, Sydney, NSW 2006, Australia

## Abstract

**Background:**

Having a ‘theory of mind’, or having the ability to attribute mental states to oneself or others, is considered one of the most central domains of impairment among children with an autism spectrum disorder (ASD). Many interventions focus on improving theory of mind skills in children with ASD. Nonetheless, the empirical evidence for the effect of these interventions is limited. The main goal of this study is to examine the effectiveness of a short theory of mind intervention for children with ASD. A second objective is to determine which subgroups within the autism spectrum profit most from the intervention.

**Methods:**

This study is a randomized controlled trial. One hundred children with ASD, aged 7 to 12 years will be randomly assigned to an intervention or a waiting list control group. Outcome measures include the completion of theory of mind and emotion understanding tasks, and parent and teacher questionnaires on children’s social skills. Follow-up data for the intervention group will be collected 6 months after the interventions.

**Discussion:**

This study evaluates the efficacy of a theory of mind intervention for children with ASD. Hypotheses, strengths, and limitations of the study are discussed.

**Trial registration:**

Netherlands Trial Register NTR2327

## Background

With a recently estimated prevalence of 1 in 88 children (1 in 54 boys) [1], autism spectrum disorders (ASD) are common and lifelong neurodevelopmental disorders, defined by a triad of impairments in social reciprocity, (non-) verbal communication and restricted and repetitive behaviors [2]. A core deficit in individuals with ASD is their limited perspective-taking, or ‘theory of mind’, ability. ‘Theory of mind’ (ToM) refers to having the ability to attribute mental states, such as intentions, beliefs, desires, and emotions, to oneself and to other people [3]. While a great many studies have shown a deficient ToM in children with ASD [4,5], it has also been shown that some individuals with ASD do develop ToM skills, albeit in a delayed fashion [6]. Whether deficient or delayed, limited ToM skills seriously impair everyday social interactions. This study examines the effectiveness of a ToM treatment in children with ASD.

Many interventions have focused on social skill development in individuals with ASD [7], with various interventions specifically focusing on ToM [8-12]. However, the empirical evidence for the effectiveness of these interventions is limited and inconclusive [13], and many studies were hampered by small samples, absence of randomized controlled trials (RCTs), and outcome measurements lacking sensitivity [14]. To date, only two RCTs have targeted the effect of ToM-focused interventions in children with ASD. First, Fisher and Happé [10,15] studied the effectiveness of the ‘picture-in-the-head’ training method in 27 children, aged 6 to 15 years, with ASD with varying cognitive abilities. This 5 to 10 day individual intervention showed increased ToM understanding in an intervention group compared with a control group, even at follow-up periods of between 6 and 12 weeks later, but no differences were found for teacher-reported ToM in everyday life. Second, Begeer *et al.*[12] studied 40 children aged 8 to 13 years with ASD without cognitive delay (IQ of 70 or above). Participants took part in a group intervention, including 16 one-hour sessions. Parents were involved to stimulate generalization of the trained skills. The intervention improved children’s understanding of beliefs, false beliefs, mixed emotions, and complex emotions. However, no impact was found on self-reported empathy or parent-reported social behavior. Both RCTs showed modest effects of the ToM intervention on conceptual understanding of ToM, whereas no results were found on practical social behavior skills as reported by parents and teachers.

These intervention studies are limited for various reasons. The sample sizes are relatively small (27 and 40 participants, respectively), resulting in insufficient power to detect subtle treatment effects. Furthermore, children with ASD may vary widely in IQ, social interaction style [16,17], the severity of the disorder [18] and comorbidity [19]. These factors may influence the impact of the intervention. In addition, previously used social skills questionnaires [10,15] focused on broad domains of behavioral inclinations (for example, the Children’s Social Behavior Questionnaire (CSBQ) [20]; the Theory of Mind and Executive Function Questionnaire [15]). It is likely that this decreased the sensitivity to subtle behavioral changes. Examining the occurrence of explicit treatment-related behavior over a limited time (for example, one week) may improve the detection of behavioral change. Finally, previous studies included either parent or teacher informants, but never both in the same study, thus ignoring different levels of structure in school or home environments, which may demand specific social skills from children.

**Figure 1:**
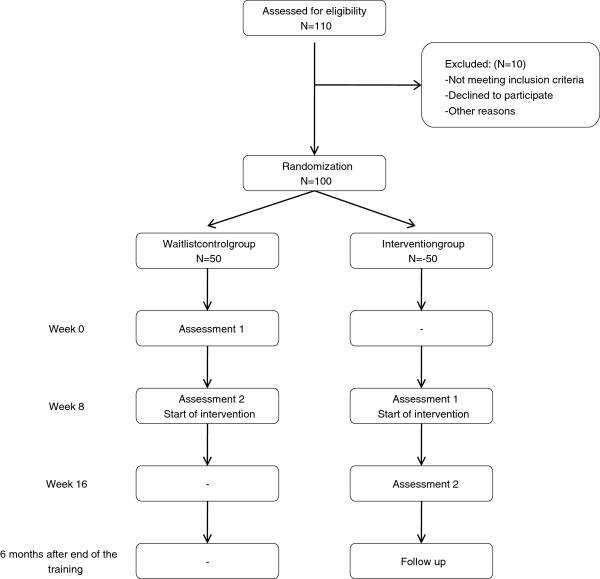
Different stages of the research procedure.

In this RCT, we examine the effectiveness of a theory of mind intervention in children with ASD and normal IQ, improving our previous study [12] on six domains (Figure 1): (1) the intervention is shortened (8 instead of 16 sessions), and (2) a larger sample is included in the trial (100 rather than 40 participants), (3) moderating and mediating variables are studied by examining the influence of social interaction style [17] and disruptive behavior [19,21] on the effect of the intervention, controlling for intellectual abilities, (4) a larger range of informants, including children, parents, and teachers, is examined with (5) more sensitive outcome measures, and (6) the persistence of the treatment effects is studied after a period of 6 months.

## Methods

### Study design

The study is a randomized controlled trial. There are two groups: an intervention group and a waiting list control group. The Medical Ethics Committee of the VU University Medical Center has approved the project (project no. 2010/241).

### Inclusion and exclusion criteria

Participants are children of 7 to 12 years old who meet the diagnostic criteria for an autism spectrum disorder (including autistic disorder, Asperger’s syndrome, and pervasive developmental disorder not otherwise specified (PDD-NOS)), according to the *Diagnostic and Statistical Manual of Mental Disorders* (DSM) [2]. This diagnosis is based on multiple assessments, including both observations and parent interviews, psychiatric and (neuro-) psychological examinations by multiple experienced clinicians (psychiatrists, psychologists, and educationalists). Additional diagnostic information is obtained by the Social Responsiveness Scale (SRS) [22]. Children are excluded if their total score is not in the clinical range. All participants have an IQ score within the normal range (70 or above). Before joining the study, both parents give active informed consent.

### Procedure

Participants are recruited from the Bascule, an academic center for children and adolescent psychiatry in Amsterdam, the Netherlands. Approximately 270 children each year are referred to the center’s outpatient clinic for ASDs.

After obtaining informed consent from parents, children are randomized to an intervention or a waiting list control group. Importantly, children in both groups start their intervention 10 weeks after the moment of randomization. The children in the control group have their first assessment directly after the randomization, followed by a waiting period of 8 weeks. Their second assessment is one week prior to the start of the intervention. The children in the intervention group have their first assessment about 8 weeks after the moment of randomization, in the week directly prior to the start of their intervention. Their second assessment is immediately after the intervention. We collect follow-up data six months after ending the intervention.

### Randomization

Randomization takes place 9 to 10 weeks before the start of the intervention. An independent research colleague randomizes the children using a digital random number generator. The randomization outcome is shared with the primary investigator, who informs parents about the condition.

### Sample size

The sample size is based on the minimum effect size (Cohen’s *d* = 0.25) found in the intention-to-treat sample of the previous trial [12]. Based on an *α* of 0.05 and a power of 0.80, we need 40 subjects in each condition to demonstrate this effect size. The additional 20 participants allow for the analysis of the effects of the two moderator variables (social interactive style and disruptive behavior).

### Intervention

The ToM intervention is a manualized weekly cognitive behavioral group intervention, including eight sessions of approximately 1 hour, provided to five or six children simultaneously, with a mutual age difference that does not exceed 3 years. Each session will be supervised by one or two certified therapists. The program is based on the ToM intervention developed by Steerneman [23], which included 200 optional exercises. This intervention was initially modified to a 16 session program [8,12]. For the current intervention, it was shortened to eight sessions. It is referred to as the ‘Mini ToM intervention’. The program is shortened for several reasons. While the 16-session program devoted relatively much attention to the introduction of basic emotions, most participating children already performed at ceiling level on basic emotion understanding tasks [12]. In the process of shortening the program, it was made sure that all the substages of the ToM were clearly represented in the intervention, but were balanced, while redundant repetition was prevented. Furthermore, in other domains of functioning, recent studies have demonstrated positive effects of both short-term and long-term interventions in autism [24]. Finally, the shorter intervention is likely to be more cost-effective and allows us to enroll more children in intervention studies. In this way, moderators of the intervention efficacy can be examined, aiming to determine which children benefit most from the intervention.

In general, the sessions all follow the same structure: (1) discussing the homework assignment, (2) games and exercises related to the day’s theme, (3) children summarizing the session to their parents, (4) explanation of the next week’s homework assignment. In the first session, the participants and the intervention are introduced. It is explained to the children that they will learn to perceive situations and people around them and that they will learn to adapt their behavior. Two different games allow the children to practice looking carefully at each other. Homework includes answering questions about the names of participants and trainers. In the second session, children are taught that what people like or dislike can differ from person to person. As assignments, they are asked to decide on activities for their group members and discuss gossiping, based on a short story, and learn to see objects from different perspectives (that is, through the eyes of an elephant and a mouse). Homework includes drawing the same object from different angles. In the third session, intentions play a central role. It is explained that it is frequently possible to predict people’s future actions based on their intentions. A fairy tale is read aloud and the children are questioned about intentions, thinking, feeling, and whether they can predict behavior, and children participate in a game about the differences between literal and figurative language. Homework includes reading a story with one of the parents or caregivers and answering questions about intentions, thinking, and feeling. The fourth session is about the emotions happiness, sadness, anger, and anxiety. It includes examples of facial expressions, body signals, and both hypothetical and personal, real-life examples. A collage is made of the four emotions and children are engaged in role-plays. In the homework assignment, children are stimulated to observe these emotions in their own environment. The fifth session continues with more complex emotions, for example, guilt, disappointment, and shame. After discussing personal experiences, a board game is played, in which children answer questions about emotions. In the homework assignment, children are stimulated to observe complex emotions in their own environment. In the sixth session, the children engage in a play, focusing on the difference between real and pretend, and taking the perspective of another person. The play is videotaped and shown to the children and parents. In the homework assignment, children need to interview their parents about their interests. In the seventh session, stories are read about protagonists who need to put themselves into the position of someone else (first-order belief ToM). For the homework assignment, children answer second-order belief questions about a story. In the last session, the children perform in a play about a family making a trip to the zoo. At the end of the intervention, each child receives a certificate.

### Measures of primary outcomes: conceptual skills

#### Theory of mind (ToM) test

The ToM test [25] is a standardized interview for children aged 5 to 13 years. It measures theory of mind knowledge and differentiates between three stages, with cognitive substages within each stage including perception and imitation, emotion recognition, elementary theory of mind, second-order belief understanding and understanding of complex humor. Children listen to a hypothetical story or look at a picture and answer the corresponding question. The test contains 72 items, which are scored on a 2-point scale (0 = incorrect, 1 = correct). Concurrent validity of the ToM test with traditional ToM tasks is moderate to high (*r* between 0.37 and 0.77) [25] and the test-retest reliability is high (intraclass correlation between 0.80 and 0.99) [12].

#### ToM advanced test

The ToM advanced test is a shortened version of ‘Stories from Everyday Life’ [6,26-28]. The test measures five forms of advanced theory of mind: understanding of second-order false belief, emotional display rules, violation of social rules, double bluff, and sarcasm. The children listen to a story and answer questions about mental states. Each mental state question is scored on a 3-point Likert scale (0 = incorrect, 1 = correct but not complete, 2 = correct). The interrater reliability of the mental state questions was good to very good, with *κ* values ranging from 0.57 to 1.00.

#### Levels of Emotional Awareness Scale for Children (LEAS-C)

The LEAS-C [29] is a questionnaire to assess children’s emotional awareness. It contains 12 scenarios describing hypothetical social situations. Children are asked about how they would feel in the described situation. Children’s use of complex emotions (for example, guilt or embarrassment) and double perspective (highlighting both own and other person’s feelings) are coded. They can attribute these emotions to themselves (one point), the other person (two points) or to both (three points). Internal consistency has been shown to be moderate (α ranging from 0.64 to 0.71) and convergent validity acceptable [12,29].

### Measures of primary outcomes: practical skills

#### Social Skills Questionnaire (SSQ)

Parent (SSQ-P) and teacher (SSQ-T) versions of the SSQ were used [30]. This is a widely used questionnaire designed to assess parents’ and teachers’ perceptions of the child’s social skills. The questionnaire contains 30 items, scored on a five-point Likert scale (0 = never true, 5 = always true). Internal consistency for the SSQ has been shown to be good, with a Guttman split-half reliability of 0.90.

#### Specific Social Behavior (SSB) questionnaire

The SSB questionnaire is a parent questionnaire designed specifically for this study to tap parents’ observations of specific theory of mind-related social behaviors displayed by their children. It contains eight items, scored on a five-point Likert scale (0 = never, 5 = always), that were based on information provided by parents during meetings set up to evaluate the intervention. Parents are asked to rate the frequency of specific theory of mind-related social behaviors.

### Measures of moderating variables

#### Wing Subgroups Questionnaire (WSQ)

The WSQ [31] is a parent questionnaire to determine the Wing social subtype of a child with ASD (aloof, passive, or active-but-odd). The WSQ contains 13 groups of four different descriptions of social behavior; each of these four descriptions characterizes one of the three Wing subtypes or a normal response. Parents evaluate how well each description fits their child on a seven-point Likert scale ranging from 0 (never) to 6 (always). Parents also choose one of each four descriptions that fit their child best. The internal consistency of the WSQ is moderate to good [17,31,32].

#### Disruptive Behavior Disorders rating scale (DBD)

The DBD rating scale [33] is a parent questionnaire to assess the symptoms of a disruptive behavior disorder in children between 6 and 16 years old. The DBD rating scale contains 42 descriptions of behavior, distinguishing between four subscales: attention deficits (9 items), hyperactivity or impulsivity (9 items), oppositional-defiant disorder (8 items), and conduct disorder (16 items). Parents evaluate how well each description fits their child on a four-point Likert scale from 1 (not at all) to 4 (a lot). Adequate psychometric properties of the DBD have been reported [33].

### Measures of control variables

#### Peabody Picture Vocabulary Test – III-NL (PPVT)

The PPVT [34] is a receptive language and screening test for verbal comprehension. It is highly correlated with a more general measure of verbal IQ, the WISC-III verbal IQ [35]. The test contains 17 sets with 12 items each, the child’s age determines the set at which the child starts. With nine or more incorrect items in one set, the test ends. The children are shown four pictures and read one word describing one of the pictures. Children need to identify the corresponding picture. A verbal IQ standardized score for age is then obtained. The PPVT has been found reliable and valid [36].

#### Social Responsiveness Scale (SRS)

The SRS [22] is a parent questionnaire designed to assess autistic traits. The SRS contains 65 descriptions of a child’s behavior, which are arranged in five subscales: social awareness, social cognition, social communication, social motivation, and autistic manners. Parents evaluate how well each description fits their child on a four-point Likert scale ranging from 0 (never true) to 3 (almost always true), and item scores are added to subscale scores. Good reliability and validity have been reported [22].

### Statistical analysis

Baseline differences in demographic and clinical characteristics will be investigated using chi-square tests and analyses of variance. To test intervention efficacy analyses of variance will be performed to compare differences between intervention group and waiting list control group in change in conceptual and practical skills. Between-group effect sizes will be calculated according to Cohen’s *d*. To test moderating effects of social interaction style (SIS) and disruptive behavior (DB) Group × SIS and Group × DB effects on change in conceptual and practical skills will be tested using multiple regression analyses, controlling for the severity of the autistic disorder and intellectual ability.

## Discussion

This study will examine the effectiveness of a theory of mind intervention for children with ASD. By comparing a randomized intervention and a waiting list control group we aim to determine whether this widely used short intervention is effective in promoting children’s conceptual and practical social skills. It is hypothesized that the intervention will increase daily life social skills of the participants, in comparison with the waiting list control group [12,15]. However, by using more sensitive measures of practical daily life social skills we also expect the intervention group to improve in their parent- and teacher-reported social behavior.

Besides determining whether this intervention is effective, a second important goal is to assess for whom this intervention is most effective. By including an adequately large sample, it is possible to address potential origins of individual differences in response to the intervention. Specifically, we aim to determine the impact of social interaction style and disruptive behavior on the intervention effect. The phenotypical heterogeneity of children with ASD requires individual care, tuned to the specific needs of each individual. By determining the influence of individual characteristics of the child on the efficacy of the current intervention we contribute to this need.

Scheeren *et al*. [17] studied social interaction styles of children and adolescents with ASD and concluded that children with specific Wing subtype interaction styles may profit from different elements of the therapy. For example, children with an active-but-odd interaction style might be expected to profit more from interventions emphasizing conceptual understanding, while children with passive or aloof styles might profit more from interventions emphasizing motivation for social interaction. Antshel *et al*. [19] highlighted the negative impact of disruptive behavior on the effectiveness of a social skills intervention in children with ASD. In this study, comorbid disruptive behavior probably also prevents children from taking full advantage of the intervention.

A limitation of the current design is the absence of diagnostic instruments, such as the Autism Diagnostic Observation Scale (ADOS [37]) and the ADI-R (Autism Diagnostic Interview-Revised [38]). However, we do rely on an extensive clinical assessment (see Participants section), and administer the Social Responsiveness Scale [22] to validate the diagnosis.

Strengths of the study include its large sample size, allowing the examination of predictors and moderators of intervention effects, and the multi-informant design, including children, parents, and teachers. Moreover, specific conceptual and practical social behavior skills are assessed as outcomes and follow-up data will be collected six months after ending the intervention. The current intervention has been used in Dutch clinics for nearly two decades [23], while similar interventions are commonly used worldwide for children with ASD. Given the limited evidence for its effectiveness, a thorough evaluation is sorely needed.

## Trial status

At the time of submission, 80% of the participants have been included in the treatment study, and pretested. Of these participants, 70% were also post tested.

## Abbreviations

ASD: autism spectrum disorders; CSBQ: Children’s Social Behavior Questionnaire; DB: disruptive behavior; DBD: Disruptive Behavior Disorders rating scale; DSM: *Diagnostic and Statistical Manual of Mental Disorders*; IQ: intelligence quotient; LEAS-C: Levels of Emotional Awareness Scale for Children; PDD-NOS: pervasive developmental disorder not otherwise specified; PPVT: Peabody Picture Vocabulary Test; RCT: randomized controlled trial; SIS: social interaction scale; SRS: Social Responsiveness Scale; SSB: specific social behavior; SSQ: social skills questionnaire; ToM: theory of mind; WISC-III: Wechsler Intelligence Scale for Children; WSQ: Wing Subgroups Questionnaire.

## Competing interests

The authors declare that they have no competing interests.

## Authors’ contributions

SB, FB, and HMK obtained funding for the study. SB, EH, CG, PC, and FB contributed to the design of the study. EH and CC coordinated the recruitment of participants. EH wrote the manuscript, in collaboration with SB and HMK. All authors read and approved the final manuscript.
